# Molecular Hydrogen in the Treatment of Respiratory Diseases

**DOI:** 10.3390/ijms26094116

**Published:** 2025-04-26

**Authors:** Dominika Zajac, Monika Jampolska, Piotr Wojciechowski

**Affiliations:** Department of Respiration Physiology, Mossakowski Medical Research Institute, Polish Academy of Sciences, 02-106 Warsaw, Poland; mjampolska@imdik.pan.pl (M.J.); pwojciechowski@imdik.pan.pl (P.W.)

**Keywords:** molecular hydrogen, respiratory diseases, lung injury, asthma, COPD, COVID-19, lung cancer

## Abstract

Molecular hydrogen is gaining increasing attention as an antioxidant, anti-inflammatory, and antiapoptotic agent. Once considered an inert gas, it reveals current therapeutic potential among others in inflammatory diseases, cancer, and sports medicine, among others. The present review aims to provide a consistent summary of the findings of the last twenty years on the use of molecular hydrogen in major respiratory diseases, including allergies, asthma, COPD, pulmonary fibrosis, lung injury of various origins, as well as cancer and infections of the respiratory tract. In addition, the basic mechanisms through which molecular hydrogen exercises its biological activity on the respiratory system are described.

## 1. Introduction

Molecular hydrogen is gaining increasing attention in modern medicine for its potential in the treatment of various diseases. It possesses unique antioxidative properties [1] not only as a highly selective free radical scavenger [2,3], but also as an inducer of endogenous detoxifying enzymes operating at the level of key transcription factors. These broad antioxidant properties contribute to its anti-inflammatory and antiapoptotic activities, which may alleviate many symptoms associated with various diseases in animal models and human therapy [4,5,6]. Molecular hydrogen, a theoretically inert gas, has emerged as a promising therapeutic agent with a wide range of potential applications.

Hydrogen (H_2_) is a colorless, odorless, and tasteless gas with poor water solubility. It reaches a maximum level of about 0.78 mM (≈1.6 mg/L) at room temperature with a loss of about 2–5% per 3 min [7]. Under normal conditions, its reactivity is negligible due to its high dissociation energy, which classifies it as an inert gas. Consequently, it is approved as a food additive in the European Union [8]. It is produced (and consumed) by bacteria of the gut microbiota [9,10] thought the degradation of oligosaccharides and mammals lack of specific enzymes (hydrogenases) required for this process. The most prominent bacterial phyla involved in this process are the *Firmicutes* and *Bacteroidetes* phyla, which include the anaerobic Clostridium species [9,10,11,12].

Following its discovery by Antoine Lavoisier in 1783, hydrogen has long been considered an inert gas without significant biological activity. This perception changed in 1888, when H_2_ was utilized for the first time as a diagnostic tool for visceral pain for the first time [13]. It was not until 1944 that hydrogen mixed with oxygen at a ratio of 96%-to-4%, known as the Hydrox gas mixture, was used by deep-sea divers to prevent decompression sickness and allow diving to depths of up to 500 m [14].

Later, in 1975, the initial observations on the possible use of hyperbaric hydrogen in the treatment of cancer in animals were conducted [15]. Despite these observations, molecular hydrogen remained neglected until the beginning of the 21st century, when the first publications on the anti-inflammatory [16] and antioxidant [3] properties of H_2_ were released, and a new era of the application of molecular hydrogen in medicine began.

Since that time, several hundred publications have been published exploring the use of molecular hydrogen in various fields of medicine, veterinary sciences, and human wellness. These studies cover a wide range of topics, including adjunctive therapies for inflammatory diseases, cancer treatment, and even preventing muscle fatigue in athletes, improving skin quality, or curing common hangovers. During the recent COVID-19 pandemic, H_2_ has received considerable attention as a potential therapeutic agent.

**Figure 1:**
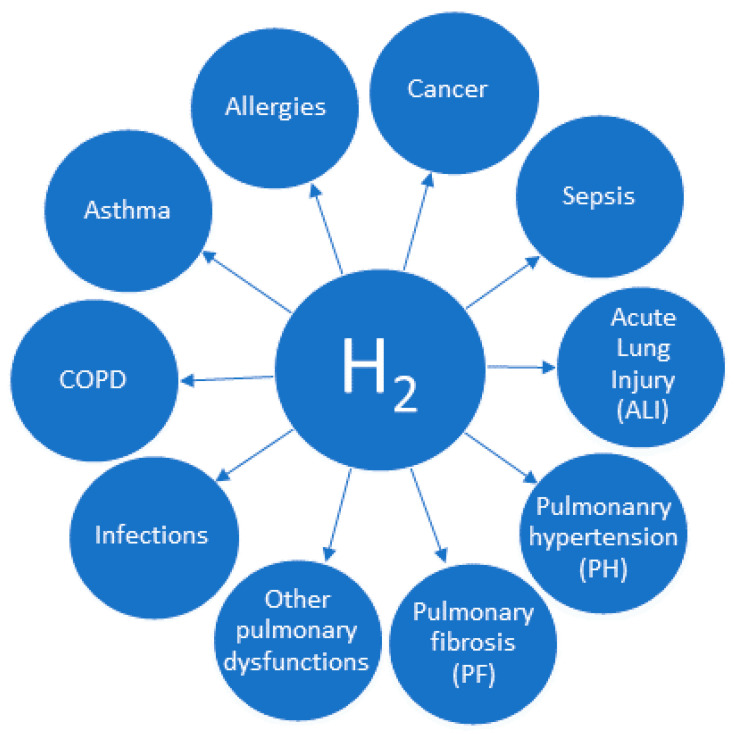
The most important respiratory disorders with proven activity of molecular hydrogen. Major respiratory disorders with proven therapeutic efficacy of molecular hydrogen (H_2_), including pulmonary fibrosis (PF), pulmonary hypertension (PH), sepsis, acute lung injury (ALI), and other conditions such as asthma, COPD, or cancer.

Over the past two decades, many excellent reviews have been published that focus on the potential mechanisms of H_2_ action [1,3,5,6] and the potential application of molecular hydrogen in various diseases [4,17,18,19,20,21]. Most reviews on respiratory diseases include studies on the application of molecular hydrogen in one specific disease: cancer [22,23,24,25,26,27,28], sepsis-related lung injury [29,30] or COVID-19 [31,32]. There is only one review that focuses on several respiratory diseases [33]. Our article, on the other hand, collects and describes research on the use of molecular hydrogen not only in the most common lung diseases—asthma, chronic obstructive pulmonary disease (COPD), lung cancer, and lung injury—but also on allergies and respiratory infections (Figure 1). In addition, this review has been updated with studies not included in the mentioned article [33] and also recent research.It also provides a brief summary of the mechanisms of the action of H_2_ related to the respiratory system. The present review covers animal studies, including preliminary studies, reviews, research papers performed solely on cell cultures, case reports, observational studies, and clinical trials. A number of studies lack conclusive results or statistical power. Therefore, they should be treated as preliminary results or observations that inspire other researchers to conduct further research.

## 2. Mechanisms of Action of Molecular Hydrogen

### 2.1. Anti-Oxidant Activity of Molecular Hydrogen

**Figure 2:**
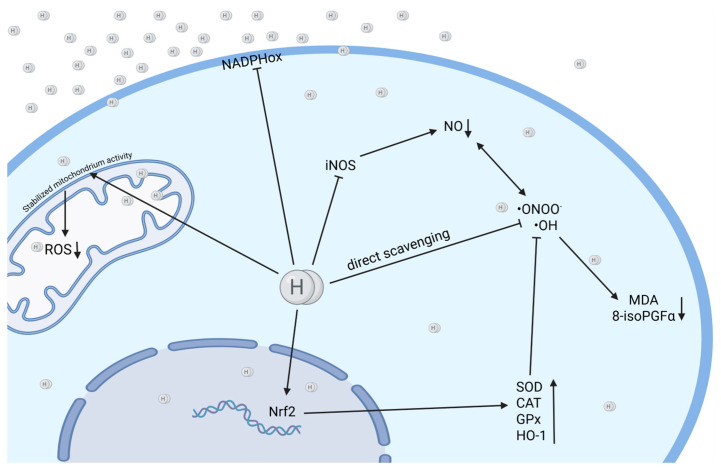
Molecular hydrogen (H_2_) may exert cyto-protective effects through direct ROS scavenging, stabilization of mitochondrial activity, upregulation of protective enzymes (SOD, HO-1) and antioxidant pathways, as well as through the downregulation of enzymes generating ROS (NADPHox, iNOS). H_2_ presents a dual mechanism—direct scavenging and enzymatic regulation (Created in BioRender. Pan, I. (2025) https://BioRender.com/qbvn8pb accessed: 11 April 2025).

The antioxidant activity of H_2_ is based on two processes: a direct scavenging of the most toxic reactive oxygen and nitrogen species (ROS/RNS), the hydroxyl radical (•OH) and peroxynitrite (ONOO^−^), and an indirect modulation of detoxifying enzymes and pathways (Figure 2).

The hydroxyl radical, a particularly potent ROS, exhibits a reactivity approximately 100 times greater than that of superoxide (O_2_^−^) [25]. It has been demonstrated to oxidize DNA and to induce lipid peroxidation processes, which result in the production of oxidative stress markers such as 4-hydroxyl-2-nonenal (4HNE), malondialdehyde (MDA) [34], and 8-Hydroxydeoxyguanosine (8-OHdG), a marker of oxidative DNA damage [35].

Peroxynitrite is one of the most potent RNS involved in nitrosative stress processes, including nitration of tyrosine residues within proteins. This process leads to the subsequent deterioration of protein function, resulting in cell necrosis or apoptosis [36].

Molecular hydrogen has been shown to selectively neutralize both •OH and ONOO^−^ without any reactivity towards other biologically active radicals such as the superoxide anion (O_2_^−^) and hydrogen peroxide (H_2_O_2_) [1,3]. Consequently, H_2_ does not disrupt physiological metabolic oxidoreduction reactions.

A significant domain of molecular hydrogen’s activity relates to its role in the maintenance of mitochondrial membrane potential, in part by reducing oxidoreductase activity, thereby preventing its dissipation. This, in turn, leads to the preservation of mitochondrial cytochrome C content [21,37]. In addition, as H_2_ diminishes the levels of mitochondrial ROS, it impedes electron leakage in the electron transport chain, modulates the mitochondrial respiratory chain complex I (though not complex II) [38,39], and increases the expression of mitofusin-2 (a protein essential for mitochondrial fusion) [40] while decreasing the levels of the dynamin-related protein 1 (Drp1 protein), a regulator of mitochondrial fission) [41,42]. Consequently, this results in the restoration and subsequent regulation of mitochondrial dynamics and function [41,43]. In parallel, hydrogen has been shown to inhibit mitochondrial stress and modulate mitophagy [44].

In addition to the direct neutralization of •OH and ONOO^−^, which results in the protection of the genome from oxidative damage, H_2_ also activates a variety of detoxifying enzymes. These include the glutathione/thioredoxin system [8], superoxide dismutase (SOD), catalase (CAT), and hemoxygenase-1 (HO-1), which are key antioxidant enzymes [5,8,21,45,46,47,48,49].

Furthermore, additionally, H_2_ activates the Nrf2 (nuclear factor erythroid 2-related factor 2) pathway, a key transcription factor involved in oxidative stress-related responses, including cytoprotective, antioxidant, and detoxifying enzymes such as HO-1 [50]. Concurrently, the Nrf2 pathway plays a pivotal role in respiratory diseases and links oxidative stress and inflammation [51]. It is noteworthy that HO-1, which is activated via the Nrf2 pathway, plays a pivotal role in maintaining the balance between pro- and anti-oxidative, pro- and anti-inflammatory, and pro- and antiapoptotic processes within the lung [52]. The protective effects of HO-1 upregulation have been observed in models of lung injury and its symptoms, including pulmonary edema, inflammation, and lung cell apoptosis [53]. These effects may be attributed to the removal of free heme and inhibition of the Fenton reaction [1,52]. The Fenton reaction, in turn, has been associated with the development of symptoms of lung injury through the enhancement of oxidative stress, as it results in the production of the most toxic ROS, namely, the hydroxyl radical [25]. Prolonged or excessive oxidative stress, which cannot be counterbalanced by the endogenous antioxidant system [54], leads to oxidative damage of alveolar macrophages and epithelial cells, their apoptosis, and, thus, to induced histological changes of the lungs such as pulmonary edema. Concurrently, it has been observed to induce overactivation of proinflammatory pathways such as the NFκB pathway and thereby contribute to the development of inflammation [55]. Disturbances in HO-1 expression and its levels have been reported in several lung diseases [56]. Notably, the activation of the Nrf2 pathway has been shown to inhibit the NLRP3 (NLR family pyrin domain containing 3) inflammasome, a protein complex that plays a pivotal role in the propagation of inflammatory responses and the regulation of cell death processes [57].

Concurrently, H_2_ suppresses the activation of the NADPH oxidase pathway and downregulates the expression of NOX2 and NOX4, two subunits of NADPH oxidase responsible for the generation of ROS [5,21,58,59]. Furthermore, H_2_ has been shown to reduce the overactivation of myeloperoxidase (MPO), one of the markers of neutrophil activation and degranulation, leading to the inhibition of neutrophilic inflammation, a hallmark of several chronic respiratory diseases [21,60,61].

### 2.2. Anti-Inflammatory Activity of Molecular Hydrogen

**Figure 3:**
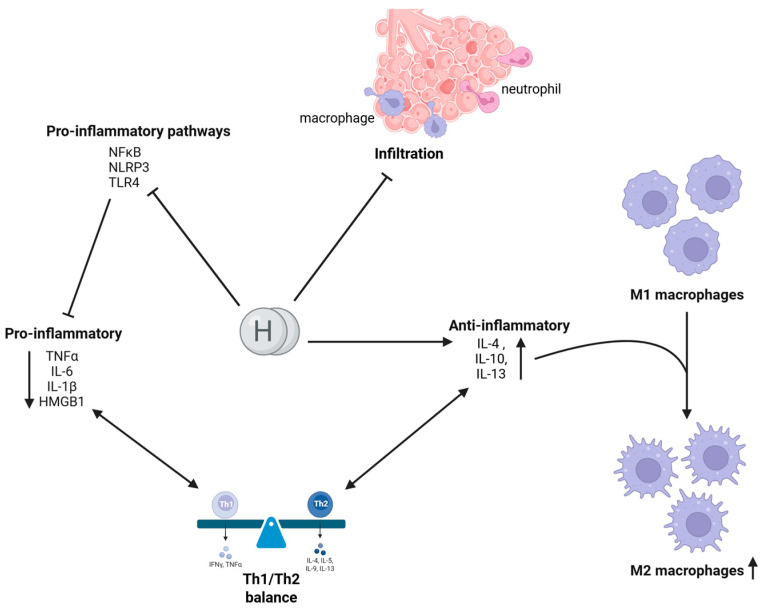
Anti-inflammatory activity of molecular hydrogen. Molecular hydrogen (H_2_) attenuates proinflammatory signaling by inhibiting NFκB nuclear translocation, blocking NLRP3 inflammasome activation, and modulating TLR4 signaling, thus limiting the release of inflammatory mediators (TNF-α, IL-6, IL-18, HMGB1). Concurrently, H_2_ promotes the production of anti-inflammatory cytokines (IL-4, IL-10, IL-13), which polarize M1 macrophages toward an M2-like phenotype. Together, these actions help restore the Th1/Th2 balance. Furthermore, molecular hydrogen mitigates macrophage and neutrophil infiltration into the bronchioles, reducing oxidative and tissue damage, preventing excessive mucus production, and ultimately preserving pulmonary function in inflammatory lung diseases. Notably, H_2_ achieves these effects without suppressing essential immune defenses, unlike conventional anti-inflammatory therapies (Created in BioRender. Pan, I. (2025) https://BioRender.com/mx9zdvq accessed: 11 April 2025).

The anti-inflammatory properties of molecular hydrogen are primarily attributed to its antioxidant activity as a scavenger of ROS and RNS (Figure 3). A decrease in the production of ROS coupled with an increase in their elimination or scavenging by H_2_ would result in the downregulation of the overactivated NLRP3 inflammasome. This, in turn, would further suppress the NFκB (nuclear factor kappa-light-chain-enhancer of activated B cells, the key transcription factor involved in oxidative stress and inflammation-related responses) pathway [4,62,63].

Concurrently, H2 exercises its inhibitory effect on proinflammatory pathways encompassing NFκB and MAPK (mitogen-activated protein kinase) cascades (such as p38, ERK, and JNK) [64]. Consequently, the ROS-dependent proinflammatory pathways like NLRP3 and TLR4 (Toll-like receptor 4), which play a role in chronic inflammation and responses to LPS (Lipopolysaccharide), respectively, are attenuated [43,63,65]. Furthermore, expression of anti-inflammatory cyto- and chemokines such as interleukin (IL)-4, IL-10, IL-13, is upregulated [5,17,43,66,67]. Conversely, proinflammatory cytokines such as IL-1β, IL-6, tumor necrosis factor alpha (TNF-α), interferon gamma (INF-γ), and high mobility group box 1 protein (HMGB1) [5,21,43,66] have been observed to be downregulated along with adhesion molecules (such as vascular cell adhesion molecule 1 (VCAM-1) or intercellular adhesion molecule 1 (ICAM-1)) [21,67,68,69,70]. In addition, H_2_ is directly involved in the regulation of signaling protein Sirtuin-1 (SIRT-1), a potential link between oxidative stress and inflammation [71]. SIRT-1 plays a pivotal role in the regulation of cell proliferation and differentiation, stress responses, and apoptosis. Additionally, it functions as a protective mechanism during acute lung injury (ALI) [72]. At the cellular level, the anti-inflammatory properties of H_2_ are manifested by decreased neutrophil and macrophage activity and migration into the injured tissue [43,68,73]. Additionally, H_2_ promotes the polarization of macrophages from the proinflammatory M1 type to the anti-inflammatory M2 type [43,74]. It also inhibits Th2 responses, restores regulatory T cells (Treg), and, thus, normalizes an overactivated immune system [21,43].

### 2.3. Antiapoptotic Activity of Molecular Hydrogen

Another category of properties that emerge from its antioxidant characteristics is the antiapoptotic one. As mentioned previously, molecular hydrogen affects a number of signaling pathways, including the PI3K/Akt/GSK3b, Ask1/JNK, JAK2/STAT3, Ras/ERK1,2/MEK1,2 pathways, all of which play a crucial role in maintaining the pro/antiapoptotic balance. Consequently, H_2_ has been shown to downregulate the expression of proapoptotic factors such as Bax and the ROS-p53 signaling pathway [29,43] and upregulate the antiapoptotic factors, including Bcl-2 and Bcl-xl. This phenomenon is likely facilitated by the activation of the PI3K/Akt and JAK2/STAT3 signaling pathways [21,43,72,75]. Concurrently, protective mechanisms at the mitochondrial level resulting from decreased ROS production and reduced oxidative stress also play a critical role in the protective effects of molecular hydrogen [5].

Furthermore, molecular hydrogen has been demonstrated to regulate endoplasmic reticulum (ER)-related stress, which is believed to be the link between ROS generation and cell death [72]. This process depends on the excessive accumulation of unfolded proteins in the ER. H_2_ has been shown to impede this process by interacting with the PERK-eIF2a-ATF4, IRE1-XBP1, and ATF6 [21]. Furthermore, it regulates autophagy, a process involved in the degradation and recycling of macromolecules. While autophagy plays a protective role under normal physiological conditions, its overactivation can lead to inflammation and tissue damage [6]. The activity of H_2_ relies mainly on the regulation of the expression of p-mTOR/mTOR and p62 proteins [17,21,65].

## 3. Methods of Administration

**Figure 4:**
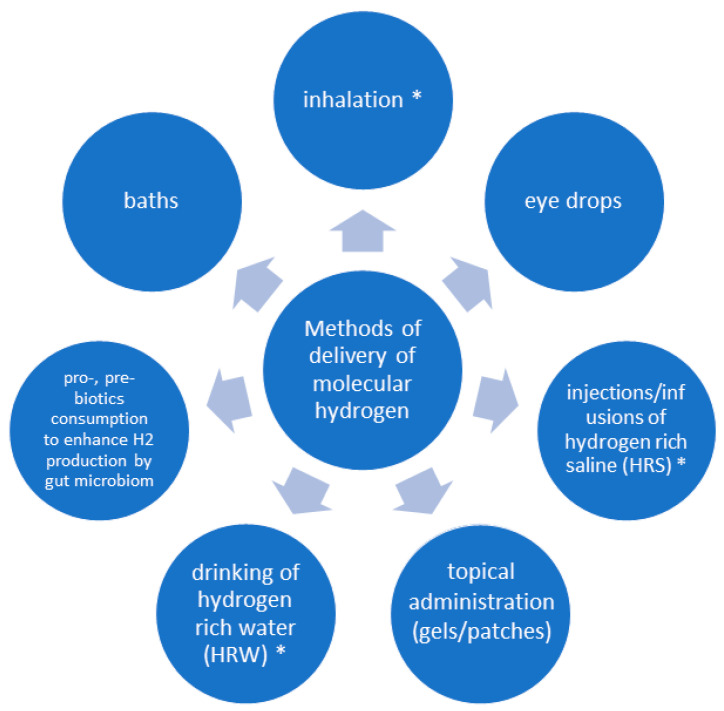
Methods of administration of molecular hydrogen (*—methods relevant for lung diseases). Methods of molecular hydrogen (H_2_) administration for therapeutic applications, with inhalation, drinking hydrogen-rich water (HRW), or infusion of hydrogen-enriched saline (HRS) being the most relevant for the treatment of lung diseases. Other approaches include topical applications (gel/patches, baths), the use of probiotics to boost the endogenous H_2_ production by intestinal microbiota, or administration to the ocular surface.

There are several methods to administer H_2_ [76], the most prominent being inhalations (2–4% in air or 66% in oxygen), the drinking of hydrogen-enriched water (HRW), and injections of hydrogen-enriched saline (HRS) [1,19] (see Figure 4). The generation of pure H_2_ typically occurs through dedicated devices that employ hydrolysis of water or solutions containing electrocatalysts such as potassium hydroxide [20,76].

HRW is intended for consumption (drinking) and can be obtained from magnesium- or calcium-containing sources [76,77]. In some Asian countries, some of them are approved as dietary supplements [78] while others, containing magnesium, citric acid, and ascorbic acid, comply with European regulations [79]. It has been found that a single calcium-containing tablet can produce up to 2.5 µg of H_2_ [80]. Additionally, another source of H_2_ includes commercially available hydrogen-generating water tanks or flasks, which are mostly based on electrolysis of filtered water [76,81].

The consumption of certain prebiotics, especially those rich in dietary fiber, indigestible starches, and sugars (lactulose), has been demonstrated to enhance intestinal H_2_ production through the activity of intestinal flora [10,28,82].

A number of alternative methods are currently under development, including the use of magnesium-hyaluronic acid complexes, palladium cells for the long-term storage of H_2_, nanobubbles, and nanotechnology-based magnesium alloys. Other methods include implantable magnesium galvanic cells for targeted delivery to tumors. Their initial testing has been performed in animal studies [49,76,83,84].

In all cases, safety issues, such as the risk of explosion at concentrations exceeding 4% in air, present concerns regarding biodegradation, and the general tolerability of the implants, as well as stability and limited storage time of the prepared H_2_-enriched solutions, should have to be taken into account.

## 4. Molecular Hydrogen and Allergies

The potential benefits of molecular hydrogen in the treatment or prevention of allergic reactions or their exacerbations remain to be fully elucidated. To date, the majority of studies on this topic have been conducted on allergy-related diseases, specifically allergic rhinitis (AR) and allergic asthma in the ovalbumin (OVA) animal models, along with observations in humans suffering from AR with and without nasal polyps. In all cases, molecular hydrogen was administered via inhalation, direct instillation into the nostrils, or as nasal lavage. All studies reported a decrease in the severity of nasal symptoms, such as itching, scratching, or a runny nose [85,86]. In addition, lower inflammatory markers, such as blood eosinophil count, levels of eosinophilic protein or mucosal eotaxin, IL-4, IL-5, IL-13, and monocyte chemoattractant protein-1 (MCP-1), were measured [86,87,88]. Concurrently, patients observed reduced tissue congestion, nasal edema, a general feeling of inflammation and sickness, and improved quality of life. At the same time, no adverse effects of H_2_ administration have been reported [89,90,91].

Specifically, molecular hydrogen has been demonstrated to restore the Th1/Th2 imbalance by downregulating Th2 cell responses, upregulating the levels of IL-10 and transforming growth factor (TGFβ) [85], and decreasing CD4+ T cell infiltration [6,92]. H_2_ increased the population of CD4+CD25+Foxp3+ Treg cells, which are often decreased in allergic rhinitis (AR) [87]. Consequently, H_2_ exerted a regulatory effect on the damaged nasal mucosa and thereby stabilized the impaired functions of macrophages [85,87].

The anti-inflammatory effects of H_2_ appear to be strongly associated with its antioxidant properties, as a parallel reduction in oxidative stress has been reported [86]. Oxidative stress, a key marker in AR and other nasal inflammatory processes, has been identified as a pivotal mediator of these pathologies. Inflammation has been shown to promote oxidative stress responses, which, in turn, exacerbate allergic inflammation [89]. Increased ROS was found in nasal polyps, and the blockade of the Nrf2 pathway in nasal epithelial cells has been shown to enhance susceptibility to sinusitis [89,93,94].

A study on type I hypersensitivity has been published by Itoh et al. [58]. In this study, H_2_ reduced mast cell degranulation via the inhibition of high-affinity immunoglobulin E receptor (FcεRI IgE) accumulation, which, in turn, led to a decreased release of histamine, leukotrienes, and cytokines, the reduced production of H_2_O_2_ by inhibiting NADPH oxidase activity, and its downstream signaling. The consumption of HRW by sensitized mice resulted in reduced vascular permeability and reduced histamine release.

A study by Choi et al. demonstrated the potential use of molecular hydrogen to prevent allergic reactions by shortening the duration of allergen exposure to the respiratory system. The study indicated that the administration of HRW led to an augmentation of elimination rates of carbon nanoparticles and a diminution of their proallergic properties, along with the pro-oxidant and proinflammatory potential of these particles [95].

## 5. Molecular Hydrogen and Asthma

Asthma is among the most prevalent inflammatory diseases of the airways. At present, approximately 300 million people worldwide are affected by this disorder [96]. The symptoms are predominantly associated with persistent airway inflammation and reversible airway obstruction, and include wheezing, dyspnea, cough, and chest tightness. At the cellular level, the symptoms are associated with an increased influx of cells into the airways, chronic oxidative stress, and an imbalance of Th1/Th2 responses, as well as pro- and anti-inflammatory cytokines, which, subsequently, result in airway hyperreactivity.

In experimental models of allergic (OVA) asthma, H_2_ administration through inhalations or drinking of HRW has been shown to ameliorate most asthma symptoms. The studies reported a decrease in airway hyperreactivity and lung resistance [33,97], airway inflammation measured as the inflammatory cell influx into the airways [74], the total number of these cells and the levels of their markers, such as MPO [33,97,98,99], and the levels of proinflammatory cytokines and chemokines (IL-4, IL-5, IL-6, IL-13, TNF-α, CXCL15) in blood and bronchoalveolar lavage fluid (BALF) [33,97,98]. In addition, goblet cell hyperplasia, mucus accumulation, and mucin-5AC (MUC5AC, the major glycoprotein of mucus [100]) expression was found to be reduced [33,74,97,98]. H_2_ administration attenuated oxidative stress expressed as lower MDA and other lipid peroxidation markers along with an enhancement in the expression and activity of endogenous antioxidant enzymes such as SOD or CAT [33,97]. Furthermore, airway remodeling, evidenced by extensive collagen I deposition and collagen III and VEGF expression, was prevented [33,98]. Epithelial barrier damage was prevented through the increased expression of E-cadherin and the tight junction protein zonula occludens-1 (ZO-1) [101], along with inhibition of proapoptotic processes like the caspase 3 and 9 pathways [99]. The phagocytic activity of macrophages was also restored [74]. Again, H_2_ inhalations or drinking had no effect on non-asthmatic, healthy mice [99].

Huang et al. described that H_2_ treatment reversed the phagocytic defect of macrophages [74]. The observed effects were attributed to the activation of the antioxidant and anti-inflammatory Nrf2 pathway, co-dominant over the inhibition of the proinflammatory NFκB pathway [74,98].

An interesting theory was proposed by Niu et al. [102]. One of the probable molecular mechanisms underlying the development and progression of asthma would be a metabolic switch from oxidative phosphorylation to anaerobic glycolysis. The study further postulates that monocytes derived from human asthmatics and lungs from OVA mice exhibit augmented lactate production and elevated glycolytic enzyme activity, consequently leading to diminished activity of mitochondrial respiratory complex I and II. This, in turn, results in impaired ATP production. The potential benefits of H_2_ administration would depend on the reversal and regulation of this metabolic switch by normalizing the increased activity of glycolytic enzymes and decreased mitochondrial enzyme activity, which would lead to the normalization of ATP and lactate levels, along with the normalization of SOD and glutathione peroxidase (GPx) activities.

There have been initial attempts to investigate the possible beneficial effects of molecular hydrogen administration in asthma. The study by Singh et al. [103] demonstrated that the drinking of HRW by patients with asthma and COPD leads to an increase in blood oxygen saturation, vitamin E levels, along with lower oxidative stress markers such as thiobarbituric acid reactive substances (TBARS), MDA, or diene conjugates. In addition, the study noted an enhancement in the patients’ exercise tolerance. Another study by Wang et al. [104] showed the beneficial effect of a 45-min inhalation of 2.4% H_2_ in air on inflammatory markers such as IL-4, IL-6 in exhaled breath condensate (EBC), and MCP-1 and IL-8 in blood.

Two other studies examined not asthma itself but concentrated rather on its common triggers of specifically occupational and air-pollution-related asthma. Choi et al. [95] demonstrated that drinking HRW and intraperitoneal (ip.) injections of HRS led to an augmentation in the elimination of carbon nanoparticles, accompanied by a reduction in lung inflammation and allergic responses following exposure. The mechanisms underlying H_2_’s activity are associated with the inhibition of lipid peroxidation via the suppression of Fenton reactions, consequently via the antioxidant properties of H_2_.

In a related study, Feng et al. [105] administered particulate matter with a diameter of less than 2.5 µm (PM_2.5_) to rats, followed by H_2_ inhalations. PM_2.5_ has been shown to react via the aryl hydrocarbon receptors, thereby increasing oxidative stress and promoting pro-oxidant and proinflammatory responses in the lungs. Consistent with the findings in other asthma studies, H_2_ inhalations were found to mitigate histopathological changes, mucus hypersecretion, and excessive mucin 5AC (MUC5AC) expression. Furthermore, H_2_ inhalations have been demonstrated to reduce oxidative stress and oxidative damage, as well as lung inflammation, as evidenced by lower levels of IL-1β, IL-8, and TNF-α. Consequently, H_2_ demonstrated a protective effect against pulmonary dysfunction and decreased lung mechanics. The beneficial effects of H_2_ can be further related to direct inhibition of aryl hydrocarbon receptors.

In summary, the findings suggest that hydrogen gas inhalations could serve as a valuable adjunct therapy during acute asthma exacerbations or in response to direct exposure to environmental triggers.

## 6. Molecular Hydrogen and COPD

Another prevalent lung disease is chronic obstructive pulmonary disease (COPD), which is characterized by progressive airway obstruction, shortness of breath, dyspnea, emphysema, and chronic bronchitis. No single cause is known, and risk factors include smoking, air pollution, and occupational exposure to dusts, chemicals, and fumes. At the cellular level, both inflammation and chronic oxidative stress play a pivotal role [106,107,108,109].

Animal models are based on the long-term exposure of rodents to cigarette smoke (CS), which is indeed the major exposure risk for the development of COPD. H_2_ has been administered by inhalations, ip. injections of HRS or drinking of HRW with similar results. In general, H_2_ administration has been found to lead to enhanced survival and reduced weight loss [110], improved lung function and static lung compliance, and decreased arterial blood pressure [110,111,112].

In addition, reduced lung damage was observed, along with ultrastructural changes such as alveolar disorders, alveolar wall thickening, ciliated cells degeneration, small airway remodeling, and emphysema, accompanied by reduced epithelial cell apoptosis [110,111,112,113]. Furthermore, a decrease in inflammation and inflammatory cell infiltration was observed [110], along with a reduction in goblet cell hyperplasia, and a CS-induced upregulation of MUC5AC expression was also documented.

Furthermore, the levels of proinflammatory cytokines (IL-6, IL-17, IL-23, Matrix metalloproteinase-12 (MMP-12)) and the expression of proapoptotic markers in lung tissue and BALF were reduced [110,111,113]. Furthermore, a tissue inhibitor of metalloproteinase-1 (TIMP-1, a pivotal factor in maintaining tissue integrity [114]) expression was elevated [110].

Observations indicated a decrease in oxidative stress, the primary pathology in COPD, accompanied by a reduction in levels of oxidative DNA damage markers [112].

At the molecular level, H_2_ decreased the CS-upregulated phosphorylation of the epidermal growth factor receptor (EGFR), but without changes in EGFR protein levels [113] along with decreased activation of the ERK1/2 and NFκB pathways [111].

In human studies, the drinking of HRW by COPD patients has been found to increase their blood oxygen saturation, exercise tolerance, and reduce oxidative stress markers and general hypoxia [103]. A double blind, parallel-controlled trial demonstrated that inhaled combination therapy comprising hydrogen (H_2_) and oxygen (O_2_) resulted in better outcomes during COPD exacerbations compared to inhaled pure oxygen alone [115]. Patients exhibited better breathless, cough, and sputum scale (BCSS) scores, suggesting a reduced severity of COPD exacerbations. However, no significant differences were observed in pulmonary function or blood gas parameters. In a separate study, COPD patients inhaled 45 min of XEN gas (2.4% H_2_ in air), and even such a brief inhalation period led to a reduction in inflammatory markers in EBC and blood [104].

In conclusion, the beneficial effects of molecular hydrogen are related to its antioxidant and anti-inflammatory properties, suggesting it to be a useful adjunctive therapy during COPD exacerbations.

## 7. Molecular Hydrogen and Pulmonary Fibrosis

Another lung disease in which molecular hydrogen has been shown to be beneficial is pulmonary fibrosis (PF), a type of interstitial lung disease characterized by fibrotic changes within the lungs and a progressive and irreversible decline in lung function. It has been associated with overactivation of myofibroblasts originating from the epithelium via the epithelial-to-mesenchymal transition (EMT), which, when prolonged, leads to the destruction of alveolar-capillary units and excessive extracellular matrix deposition. This process contributes to structural remodeling and pulmonary fibrosis. EMT is often initiated by excessive oxidative stress, which activates the transforming growth factor TGF1β via an ROS-dependent pathway [41].

Animal models using different fibrosis-inducing agents (paraquat, LPS, or bleomycin) have shown that the administration of H_2_ by inhalations or drinking (HRW) has been shown to reduce lung stiffness and preserve the lung’s ability to expand, along with an augmented lung capacity [116,117]. At the cellular level, decreased inflammation (IL-4, IL-6, IL-13), oxidative stress (ROS, MDA, hydroxyproline), and fibrosis (fibronectin, collagen deposition) were observed [116,117,118,119]. Furthermore, a decrease in M2 macrophages and EMT, along with reduced α-smooth muscle actin (α-SMA) and TGF1β expression in the lungs, has been described. The potential mechanisms underlying these processes involve the H_2_’s ability to reduce oxidative stress, diminish p38/MAPK and JNK pathway activation, and attenuate general inflammation, thereby modulating fibrotic responses [33].

## 8. Molecular Hydrogen and Other Pulmonary Diseases

Molecular hydrogen has been utilized in the treatment of other, less prevalent pulmonary diseases. Inhalations of a 2:1 ratio of hydrogen to oxygen (H_2_:O_2_) have been demonstrated to reduce airway resistance and inspiratory effort in patients with tracheal stenosis [120]. In a separate study, mice with obliterative airway disease exhibited reduced airway occlusion, increased CD4+/CD3+ ratio, decreased IL-6, and increased Treg activity after drinking HRW, with no significant impact on the Nrf2 pathway [121].

Muramatsu et al. [122] demonstrated that even prenatal exposure to H_2_ can influence the neonate’s lung function. In a mouse model of pulmonary dysplasia, LPS injections into the amniotic fluid during pregnancy resulted in an oxidative stress-dependent arrest of alveolar development in the offspring. The drinking of HRW by pregnant or feeding mice led to the normalization of the abnormally enlarged alveoli, the reduction in oxidative stress markers such as nitrotyrosine and 8OHdG, and the augmentation of LPS-suppressed expressions of HO-1, fibroblast growth factor receptor 4 (FGFR4), and vascular endothelial growth factor receptor 2 (VEGFR2), the latter being essential for proper maintenance of alveolar structures and is involved in proper lung development [123].

Similar observations have been documented by Hattori et al. [124]. Maternal infections frequently result in preterm birth and severe respiratory impairment in the neonate. In the present study, pregnant rats were injected ip. with LPS and received HRW orally. The maternal intake of HRW resulted in enhanced neonatal lung function, morphology, and developmental status, as evidenced by higher arterial blood pO_2_ and pH and lower pCO_2_. Furthermore, a reduction in the number of caspase-3-positive cells and IL-6 and 8OHdG levels was noticed. These observations suggest that maternal H_2_ consumption reduces apoptosis and oxidative stress-related damage of the lung and thereby enhances the lung function of the pup.

Another disease in which H_2_ may be beneficial is pulmonary hypertension, which is characterized, among others, by shortness of breath, elevated pulmonary arterial pressure, right ventricular hypertrophy, systemic inflammation, and increased oxidative and nitrative stress. The disease appears to be strongly associated with ROS, RNS, and chronic inflammation [69]. In experimental models in rats, H_2_ inhalations and HRW or HRS administration have been shown to reduce pulmonary arterial pressure, right ventricular weight and hypertrophy index, general and pulmonary inflammatory responses, macrophage infiltration, and markers of oxidative (measured as 8OHdG levels) and nitrative (measured as 3-nitrotyrosine levels) stress [69,125]. Again, these protective effects are based on the antioxidant and anti-inflammatory activities of H_2_ [33].

## 9. Molecular Hydrogen and Lung Injuries

### 9.1. Sepsis-Related Lung Injury

Sepsis, a serious condition marked by the organism’s excessive reaction to infection, can lead to various complications. Among the most severe are systemic inflammation, the so-called cytokine storm, acute lung injury (ALI), which can rapidly progress to acute respiratory distress syndrome (ARDS). This condition is characterized by excessive inflammatory responses, cytokine storm, and oxidative stress, along with dysregulated cell apoptosis and autophagy, as described by Zhang et al. [30]. Furthermore, the aforementioned processes are accompanied by a decline in lung function.

Research conducted on cecal-ligation-and-puncture (CLP)-induced murine sepsis models has demonstrated the efficacy of hydrogen inhalations and HRS injection during surgery [117]. In both cases, H_2_ increased survival rates, pulmonary function described as the PaO_2_/FiO_2_ coefficient, or blood gas parameters [126] together with a decreased severity of lung injury, pulmonary edema, and histological score, or lower neutrophil count [117,126]. At the molecular level, lower inflammatory markers such as proinflammatory cytokines (IL-1β, IL-6, TNF-α, HMGB1) and lower oxidative stress outcomes (lipid peroxidation, DNA oxidation, nitrotyrosine levels) were observed. The observed effects were mainly related to the antioxidant activities of H_2_ and its activation of the HO-1 and Nrf2 pathways and suppression of p38 and NFκB ones. [126]. Furthermore, this results in the upregulation of mitochondrial function and the activation of autophagy via the modulation of the mTOR/TFEB and PINK/Parkin phagocytic pathways in parallel with inhibition of the NLRP3 inflammasome, as has been demonstrated by Ren et al. [127]. In clinical settings, the co-administration of H_2_ has been observed to enhance the efficacy of standard treatment options [30,33].

### 9.2. General Lung Injury

One of the most important clinical problems in which H_2_ may be beneficial is lung injury and inflammation of any kind. These conditions can be classified according to their severity (measured as the PaO2/FiO2 index), duration (acute or chronic), or trigger or cause. Among these, bacterial and viral lung injury (LI), hypoxia/reoxygenation, hyperoxia, ventilator-, smoke-, burn, radiation-induced, and fetal LI are the most prominent. The main symptoms of acute lung injury (ALI) are histopathological changes within the lung with inflammatory cell infiltration, goblet cell hyperplasia, alveolar wall thickening, pulmonary and epithelial cell damage, edema, decreased lung function, and blood oxygenation (measured as PaO2/FiO2 index or blood gas levels). At the molecular level, ALI is characterized by increased levels of cytokines and chemokines, markers of oxidative stress and apoptosis, along with decreased levels of anti-inflammatory and antiapoptotic factors. Consequently, proinflammatory pathways, such as NFκB, are observed to be upregulated, whereas antioxidant pathways, such as the Nrf2 pathway, are downregulated. Consequently, ALI and its chronic form are a complex disorder of the pro- and antioxidant and pro- and anti-inflammatory balance.

There are numerous animal models of ALI, with the most prevalent being the LPS-instillation model. Lipopolysaccharide (LPS) constitutes a pivotal component of the bacterial cell wall of Gram-negative bacteria. Intratracheal (it.), intraperitoneal (ip.), or intravenous (iv.) administration of LPS in animals has been shown to reproduce the majority of ALI symptoms, including lung edema, leukocyte accumulation in the airways, severe lung inflammation, and others. The LPS-induced ALI exhibits a high degree of similarity with the human clinical picture of pneumonia- and sepsis-associated lung injury [128].

The H_2_ administration was carried out through inhalations at various concentrations (ranging from 2% to 66% in air or O_2_) or as injections of HRS at different time points.

The administration of H_2_ resulted in a near-complete abrogation of all symptoms associated with LPS-induced ALI, including the decreased lung function measured as blood gas parameters [29,65,129], PaO_2_/FiO_2_ index [67,130,131], ventilation parameters [129], and histological changes, including alveolar wall thickening [129], neutrophil influx [64,67,129,131], lung edema, increased wet-to-dry ratio, disturbed expression of aquaporin 1 and 5 [17,33,64,67,71,129,131,132], epithelial and alveolar cell damage [33,131,132], and increased protein contents in BALF [67,130]. Furthermore, H_2_ reduced elevated inflammatory markers, including IL-1β, IL-6, TNF-α, prostaglandin E2 (PGE2) [29,65,71,128,130], macrophage protein 1α 2 (MP1α), and monocyte chemoattractant protein-1 (MCP-1) [67], while increasing the levels of decreased anti-inflammatory markers such as IL-10 [133]. It also reduced the oxidative stress markers, including MDA and nitrotyrosine [64,132], as well as the production of ROS [29], and upregulated antioxidant enzymes such as HO-1 and SOD [64,130,132]. Moreover, the studies observed a decline in lung cell apoptosis attributed to the H_2_-induced decrease in the number of terminal deoxynucleotidyl transferase dUTP nick end labeling (TUNEL) (an assay for cell apoptosis [134])-positive cells and the levels of caspases-3 and 8 [17,33,64,130]. It has been demonstrated to upregulate the antiapoptotic markers such as Bcl-2 [17,29,64,67]. These alterations were associated with the suppression of the NFκB pathway in a SIRT-1-dependent way [29,130] alongside a downregulation of the signaling pathways connected to MAPK and JNK kinases [129]. Autophagy was also regulated, with the activation of the AMPK/mTOR/TFEB pathways being decreased [17,132]. Furthermore, molecular hydrogen was found to regulate the disrupted alveolar barrier permeability [133].

### 9.3. Hypoxia-Reoxygenation Lung Injury

In addition to the well-known LPS-based model of ALI, there are several types of lung injury (LI) and inflammation. One such example is the model of LI induced by hypoxia-reoxygenation (H/R), analogous to injuries observed in lung transplants and following cardiac arrest. The pathomechanisms underlying this type of LI include extensive oxidative stress, increased migration of neutrophils and M1 macrophages into the airways, lung cell apoptosis, increased production of proinflammatory markers, and severe deterioration of lung function resulting in hypoxemia and hypercapnia.

Inhalations of molecular hydrogen in the hypoxia-reoxygenation model have been shown not only to ameliorate impaired lung function but also to significantly decrease oxidative stress and inflammation [68]. At the molecular level, these changes were mainly based on the H_2_-dependent reduction in hydroxyl radical production and induction of HO-1 and SOD, which led to the subsequent inhibition of inflammatory markers such as IL-1β and TNF-α. These changes also resulted in the inhibition of the pro-oxidant and proinflammatory p38 MAPK and NFκB pathways, which occurred in parallel to the inhibition of apoptosis and pyroptosis [30,33,68].

Pretreatment of the lung graft donor by H_2_ inhalation prevented lung inflammation after H/R, as evidenced by decreased levels of IL-1β, TNF-α, ICAM-1, and reduced inflammatory cells influx, along with improved lung function, as reflected in increased arterial pO_2_ and decreased pCO_2_ [135]. Reduced histological and apoptotic changes, such as alveolar septum thickening, were observed. Further, a reduction in oxidative stress markers such as lipid peroxidation and proapoptotic markers, including Bax and caspase-3, was observed. Concurrently, an increase in antiapoptotic proteins such as Bcl-2 and Bclx was noted. [136].

Consistent observations have been made by Haam et al. [137] in H_2_-inhaling pigs with cardiac arrest. The animals showed enhanced lung function, improved blood gas parameters and diminished lung injury severity score, and lung edema during both H_2_ inhalation and reperfusion. In addition, the study noted higher levels of antioxidant enzymes (HO-1, SOD) and anti-inflammatory cytokines (IL-10) along with a reduced number of apoptotic cells.

### 9.4. Hyperoxia-Related Lung Injury

Another type of lung injury frequently observed in clinical settings is hyperoxic (hyperoxia-related) LI. Unlike hypoxia and subsequent hypoxemia, which are characterized by insufficient levels of inspired oxygen, hyperoxia is mainly related to elevated oxygen levels resulting from the administration of high-concentration or pure oxygen. Hyperoxia has been demonstrated to be closely associated with oxygen toxicity and can result in elevated oxygen stress and its associated consequences. They include lung inflammation, the overactivation of neutrophils and macrophages, edema, and, ultimately, alveolar damage and pulmonary dysplasia [75,138,139]. There have been attempts to use molecular hydrogen, predominantly administered as HRS ip., as a therapeutic intervention in the case of hyperoxia-related lung injury. H_2_ administration resulted in a reduction in lung edema, inflammatory cells infiltration, pleural fluid volume, and hemorrhage. Further, it was observed that H_2_ administration led to a decrease in inflammation, indicated by reduced levels of IL-1β and TNF-α. At the molecular level, a study by Sun et al. reported a reduction in apoptosis (described as a lower number of TUNEL-positive cells) and its associated markers (p-Akt, p-FoxO3, cyclin D1, Bcl-2) [72]. The molecular mechanisms underlying this beneficial effect of H_2_ appear to encompass its antiapoptotic activity, which is associated with the activation of the PI3K/Akt/FoxO3+ pathway. Furthermore, H_2_ protected alveolar type II cells from hyperoxia [75], as well as increased the activation of the SIRT-1 proteins. As a result, it has been observed to decrease ER stress along with the activation of the antioxidant HO-1 and Nrf2 pathways [30,52].

### 9.5. Ventilator-Induced Lung Injury

Ventilator-induced lung injury (VILI) is a significant clinical problem that has been addressed in two murine studies [140,141]. In both studies, H_2_ was administered as inhalations, resulting in decreased lung edema, the infiltration of inflammatory cells, and decreased inflammatory mediators. Concurrently, lung function and gas exchange were enhanced [141]. An intriguing mechanism proposed by Huang et al. [140] suggests a biphasic response to H_2_ with the potential for phase-dependent alterations in downstream effects. In the initial phase, which commenced about one hour after the initiation of inhalation, the treatment resulted in NFκB pathway activation, augmented Bcl-2, and diminished Bax protein expression. In the subsequent phase, H_2_ would induce a decline in NFκB activation and NFκB DNA binding, leading to a reduction in lung cell apoptosis. As in the case of other ALIs, the antioxidant properties of H_2_ play a significant role.

### 9.6. Seawater-Induced Lung Injury

It is generally accepted that one of the most effective animal models of ALI is, in addition to the LPS-based model, the model in which seawater is instilled into the airways. Both models exhibit features such as lung edema, alveolar collapse, and surfactant disruption along with hypoxemia and increased oxidative stress. Seawater-induced ALI shows symptoms consistent with those observed in drowning victims, while LPS-induced ALI is more inflammatory in nature. Notably, osmotic stress has been identified as a contributing factor in the pathogenesis of seawater-induced ALI [61,128,142]. In a study in seawater-instilled rabbits, two-percent inhalations of H_2_ resulted in a reduction in lung epithelial permeability and histopathological changes. Furthermore, markers of both oxidative stress (such as MDA levels in lung tissue) and airway inflammation (including IL-1β, IL-6, TNF-α in BALF) were reduced, as was MPO activity as a marker of neutrophilic infiltration. In addition, proapoptotic markers such as caspase-3 and, consequently, apoptosis as a process were reduced. Other observations, regarding other types of ALI, HO-1, and Nrf2, along with their respective pathways, demonstrated increased expression, indicating their role in H_2_ activity and its impact on lung injury [61].

### 9.7. Other Forms of Lung Injury

A similar set of findings has been reported for other forms of ALI, induced by triggers such as smoke, burning, or radiation. In all cases, an improvement in alveolar structure, reduced alveolar wall thickening and lower levels of proinflammatory (IL-1β, IL-6, TNF-α), proapoptotic, and apoptotic index markers along with higher expression and activity of antioxidant enzymes such as SOD have been described. Consequently, the changes resulted in reduced ROS and MDA levels, attenuated inflammation, and lung cell apoptosis, regardless of the route of administration [30,143].

## 10. Molecular Hydrogen and Infections

Little is known about the potential application of molecular hydrogen in the treatment of infectious diseases other than COVID-19. There is a paucity of information regarding its use in the most common infections of the respiratory system, including those caused by pathogens such as Streptococcus pneumoniae, *Haemophilus influenzae*, *Moraxella catarrhalis*, *Streptococcus pyogenes*, *Mycoplasma pneumoniae*, *Bordetella pertussis*, *Burkholderia pseudomallei*, *Chlamydophila pneumoniae*, *Corynebacterium diphtheriae*, *Haemophilus influenzae*, *Mycoplasma pneumoniae*, *Streptococcus pneumoniae*, *Coxiella burnetii*, and *Legionella pneumophila*. In addition, research papers addressing the use of molecular hydrogen in the treatment of viral infections of the respiratory tract (induced by rhinoviruses, adenoviruses, influenza and parainfluenza viruses, respiratory syncytial viruses, enteroviruses) are missing.

The only application of molecular hydrogen in respiratory infections is the treatment of the acute form of COVID-19 and post-COVID syndrome. The COVID-19 disease, caused by overactivation of the immune system by the severe acute respiratory syndrome coronavirus 2 (SARS-CoV-2 virus), leads to severe inflammatory pneumonia and direct alveolar injuries caused by the virus. These injuries result in decreased alveolar gas exchange, leading to hypoxemia and dyspnea [31,144]. Consequently, the development of secondary infections and the need for mechanical ventilation may occur [31,145]. At the cellular level, the SARS-CoV-2 virus activates the INF-γ pathway, leading to an overreaction of the immune system and the so-called cytokine storm, characterized by excessive secretion of proinflammatory mediators, including proinflammatory cytokines [145]. Concurrently, the inhibition of negative feedback and the amplification of feedback responses result in increased cytokine production and activation of the NADPH pathway in leukocytes. This, in turn, enhances oxidative stress via elevated levels of ROS [32,146]. COVID-19 infection is similar in its clinical presentation to sepsis, as both conditions are characterized by the rapid development of symptoms, including persistent neutrophilia [144,147].

Human studies during the recent pandemics have shown positive effects of the co-administration of hydrogen in conjunction with classical oxygen inhalatory therapy at various concentrations. In comparison with oxygen therapy alone, 4 to 66% H_2_ in O_2_ reduced the severity of the disease, dyspnea, chest distress, cough, fatigue, and the duration of hospitalization. Concurrently, the discharge rate, blood oxygen saturation, and general well-being of the patients were higher. The respiratory effects of oxygen–hydrogen co-administration are probably related to a deeper penetration of oxygen into the bronchial area, followed by decreased airway resistance, increased oxygenation, and the improvement of general respiratory function, together with a stimulation of mucus drainage [31,62,148].

At the cellular level, the effects of H_2_ inhalations are related to its antioxidant and anti-inflammatory properties [31]. Specifically, H_2_ reduced the infiltration of neutrophils and macrophages into the lung [149], thereby inhibiting their excessive activation and the subsequent release of proinflammatory mediators, including IL-1β, IL-6, TNF-α, HMGB-1, CCL, and MCP-1. In addition, H_2_ inhibited the activation of the NLRP3, NFκB, and MPO [30,31,32,150] and modulated the Nrf2 [30] pathways. Consequently, this led to the resolution of the cytokine storm, inflammation [149,151], and limitation of airway damage [32]. When administered at the onset of a mild infection, H_2_ has been observed to prevent the onset of the cytokine storm, likely through an early suppression of oxidative stress [6,31]. Notably, these effects are not attributable to direct interactions of H_2_ with the virus [31] and are independent of its variant (Omicron vs. other virus variants) [151].

In the context of the global pandemic, H_2_ inhalations have emerged as a promising therapy for the treatment of COVID-19. This approach, adapted mainly in China [149], has demonstrated significant potential in clinical trials. It is currently being explored as a safe and cost-effective treatment option. Furthermore, hydrogen inhalations have found application in post-COVID-19 respiratory rehabilitation. H_2_/O_2_ inhalations, varying in duration, have been observed to decrease symptom severity, anxiety (described as decreased fatigue assessment scale (FAS) scores). Additionally, such inhalations have been shown to improve respiratory functions (increased forced vital capacity (FVC), minute ventilation (V_E_), and forced expiratory volume in 1 s (FEV_1_)). Improved general fitness (measured as increased distance in the 6-min walking test (6-MWT)) and higher quality of life have also been documented [18,152,153].

## 11. Molecular Hydrogen and Cancer

Molecular hydrogen demonstrated efficacy in the treatment and as an adjuvant therapy for various cancers of the respiratory tract.

In vitro studies have demonstrated that an H_2_-rich medium reduced the colony size and formation of tongue cancer cells and decreased proliferation in human fibrosarcoma and esophageal cancer cells, as well as A549 cells [154,155]. Furthermore, the treatment of H1975 cells resulted in a decrease in cell viability, migration, and invasion, along with catalyzed cell apoptosis [155].

A reduction in tumor weight and size, as well as a lower number of cells of squamous cell carcinoma, was revealed by animal studies. Moreover, increased survival of mice with colorectal cancer was observed after H_2_ administration [25,156]. A549 xenografted mice exhibited diminished tumor growth accompanied by a suppression of ROS production and the progression of inflammation (measured as decreased IL-1β, IL-13, TNF-α levels), indicating the importance of the antioxidant and anti-inflammatory properties of H_2_ also in cancer treatment [25,155]. Notably, as previously reported, H_2_ administration exhibited no effect on healthy animals or non-cancerous cell lines [25,157].

However, the most compelling observations have been made in human subjects. H_2_ inhalations or the drinking of HRW have been shown to reduce tumor marker levels, tumor mass, and their formation. These interventions led to enhanced disease control, including longer progression-free periods and higher survival rates, with some cases of complete remission [22,23,25]. Patients reported improved quality of life with better physical status and fewer pulmonary symptoms [158]. In combination with conventional (such as cis-platin) and modern (including antibodies like nivolumab) therapeutics, H_2_ enhanced drug activity, resulting in enhanced outcomes and improved disease control and reduced side effects of the treatment, such as nephrotoxicity, weight loss, insomnia, pain, or hearing loss in the case of radiotherapy [23,159,160,161]. The aforementioned effects have been observed in patients diagnosed with a variety of cancers affecting the respiratory tract. The mechanisms of such activity involve a range of processes, including the inhibition of ROS production [159], the restoration of optimal levels and activity of immune cells, including NKT, NK, T cells, and the restoration of the proper immune system functionality [22,156,159]. These processes extend to the regulation of pro- and antiapoptotic pathways. As indicated by Yang et al., the latter include the activation of the ROS/NLRP3/caspase-3/gasdermin D-mediated pyroptotic pathways [162]. Additionally, the suppression of vascular endothelial growth factor (VEGF) expression and downregulation of regulators of chromosome condensation (such as the structural maintenance of chromosomes complex (SMC)-2, 5, 6) have been documented [25,155,163]. Furthermore, the effects of H_2_ result in the suppression of the overactivated Wnt/beta-catenin signaling pathways, which further leads to suppression of tumor progression [164].

Radiotherapy is a prevalent treatment option for cancer, yet it is associated with significant adverse effects. The efficacy of radiotherapy is mainly based on direct absorption of radiation energy at the level of nucleic acids, lipids, and proteins, as well as the subsequent generation of free radicals during water radiolysis [26,27]. Major adverse effects of radiotherapy include skin damage, bone marrow disturbances, immune system dysfunctions, gastrointestinal damage, and cognitive impairments, among others. In clinical settings, a range of radioprotective agents, including vitamins C, D, E, melatonin, α-lipolic acid, and N-acetylcysteine, are employed, predominantly functioning as free radical scavengers [24,165,166]. However, these agents are not without side effects. In this context, the administration of molecular hydrogen as a novel therapeutic option has been proposed as an adjunct therapy during radiotherapy.

In vitro studies have demonstrated reduced ROS production, improved oxidative stress, and apoptosis markers, together with higher survival rates of A549, HIEC, HaCaT, and BMSC cells exposed to radiotherapy in an H_2_-rich medium [167,168,169,170].

In animals, H_2_ administration in conjunction with radiotherapy has been shown to protect against immune dysfunction [92,171], acute and chronic lung [170], myocardium [172], and gastrointestinal [173] and mucosal [174] damage. Additionally, H_2_ administration has been associated with reduced incidence of dermatitis, nephrotoxicity, and weight loss [2,168]. Furthermore, it has been demonstrated to increase survival rates [175] and promote tissue recovery and wound healing [176]. Registered clinical trials and observational human studies have shown protection against bone marrow damage during modulated radiation therapy [177] and improved quality of life, including the improvement of taste disorders or anorexia when combined with H_2_ administration [178].

The radioprotective effects of H_2_ are primarily attributed to its hydroxyl radical scavenging activity [3], underscoring its antioxidant properties and antioxidant activity via decreased levels of oxidation of mitochondrial DNA and the NLRP3 cascades. The radioprotective effects of H_2_ are further enhanced by its antiapoptotic properties, which are a result of its antioxidant and anti-inflammatory properties. Furthermore, the regulation of pro- and antioxidant gene expression may play a significant role in radioprotection.

## 12. Future Perspectives and Limitations

Today, the popularity of treatments with molecular hydrogen is on the rise. This increase in popularity is observed not only in specialized medical centers but also as a component of wellness or beauty-related treatment options. The financial burden associated with acquiring professional equipment for the generation of molecular hydrogen is diminishing both in terms of purchase and rental costs. In several countries, including Japan, capsules containing magnesium for the generation of H_2_ have been introduced on the market, and H_2_ has been approved as a dietary supplement [179]. The broad range of applications and increasing popularity of hydrogen-related treatments have led to the perception of molecular hydrogen as a novel panacea. An important question is whether the administration of molecular hydrogen could serve as an in-house treatment, a form of add-on therapy for mild infections (e.g., the common cold), akin to brine inhalations [180]. Given the documented efficacy of H_2_ in the treatment of COVID-19, it is possible that it could also be effective in the treatment of other viral diseases, including influenza. Another research point is the possible bacteriostatic activity of H_2_. Some pathogens (such as Salmonella or Mycobacteria [181,182]) have been observed to utilize molecular hydrogen during the progression of infection; therefore, it cannot be used to reduce all bacterial infections and their progression. To date, there are no data on the successful use of H_2_ in the context of bacterial invasions within the respiratory tract. However, molecular hydrogen has been observed to alleviate symptoms of LPS- and sepsis-induced ALI, which can be of bacterial origin [181,182]. Also, the possible utility of H_2_ in bacterial-viral co-infection remains to be elucidated. The potential of H_2_ inhalation to prevent the exacerbations of allergies in atopic subjects, respiratory infections in prone subjects (e.g., school children during spring/autumn infection season), and its role as an immunomodulator due to its anti-inflammatory properties, remains, however, to be elucidated.

Despite the advantageous properties of molecular hydrogen, its utilization is limited both in clinical and domestic settings. The primary concern is safety since molecular hydrogen is explosive above a concentration of 4–5% in air; moreover, special equipment is required to assure safe administration above this limit, and, thus, this type of delivery is rather limited to specialized facilities. Second, the precise dosing of H_2_ is challenging, as all methods described in this review, with the exception of inhalations, fail to ensure the desired levels of H_2_. Thirdly, the stability of hydrogen-rich solutions poses significant challenges, as about 2–5% of dissolved hydrogen is lost from open containers every 3 min [8]. Consequently, there is an urgent need to develop cost-effective and efficient capture and storage systems to mitigate these losses. Another issue is the financial burden associated with the production of hydrogen, the costs of the hydrogen-generating and storage devices, and the maintenance of these systems.

## 13. Concluding Remarks

Molecular hydrogen has garnered increased attention as a relatively simple, cost-effective, and safe treatment option for acute and chronic respiratory diseases. Its multifaceted activity involving anti-inflammatory, antioxidant, and antiapoptotic mechanisms facilitates a more holistic approach to the treatment, addressing multiple facets of the disease rather than a single symptom or pathway. Consequently, there remains considerable potential for the utilization of molecular hydrogen in medical disciplines and associated domains of human and veterinary medicine.

## Data Availability

Not applicable.

## Abbreviations

The following abbreviations are used in this manuscript:

•OH	hydroxyl radical
4HNE	4-hydroxyl-2-nonenal
6-MWT	6-min walking test
8-OHdG	8-hydroxydeoxyguanosine
A549 cells	adenocarcinomic human alveolar basal epithelial cells
ALI	acute lung injury
AR	allergic rhinitis
ARDS	acute respiratory distress syndrome
BALF	bronchoalveolar lavage fluid
BCSS	breathless-cough-sputum scale
BMSC	bone marrow-derived mesenchymal stem cells
CAT	catalase
CLP	cecal-ligation-and-puncture
COPD	chronic obstructive pulmonary disease
COVID-19	coronavirus disease 2019
CS	cigarette smoke
Drp1	dynamin-related protein 1
EBC	exhaled breath condensate
EGFR	epidermal growth factor receptor
EMT	epithelial-to-mesenchymal transition
ER	endoplasmic reticulum
FAS	fatigue assessment scale
FcεRI	high-affinity IgE receptor
FEV1	forced expiratory volume in 1 s
FGFR4	fibroblast growth factor receptor 4
FVC	forced vital capacity
GPx	glutathione peroxidase
H/R	hypoxia-reoxygenation
H1975	human non-small cell lung cancer cells
H2O2	hydrogen peroxide
HaCaT	aneuploid immortal keratinocyte cell line from adult human skin
HIEC	human intestinal epithelial cells
HIF-1α	hypoxia-inducible factor-1 α
HMGB1	high mobility group box 1 protein
HO-1	hemoxygenase-1
HRS	hydrogen-enriched saline
HRW	hydrogen-enriched water
ICAM-1	intercellular adhesion molecule 1
IgE	immunoglobulin E
IL	interleukin
INF-γ	interferon gamma
ip.	intraperitoneal (injection)
it.	intratracheal (administration)
iv.	intravenous (administration)
LI	lung injury
LPS	lipopolysaccharide
MAPK	mitogen-activated protein kinase
MCP-1	monocyte chemoattractant protein-1
MDA	malondialdehyde
MMP-12	matrix metalloproteinase-12
MP1α	macrophage protein 1α
MPO	myeloperoxidase
MUC5AC	mucin-5AC
NFκB	nuclear factor kappa-light-chain-enhancer of activated B cells
NLRP3	NLR family pyrin domain containing 3
Nrf2	nuclear factor erythroid 2-related factor-2
O2-	superoxide
ONOO⁻	peroxynitrite
OVA	ovalbumin
pCO2	partial pressure of carbon dioxide
PF	pulmonary fibrosis
PGE2	prostaglandin E2
PM2.5	particulate matter of a diameter of less than 2.5 µm
pO2	partial pressure of oxygen
ROS/RNS	reactive oxygen and nitrogen species
SARS-CoV-2	severe acute respiratory syndrome coronavirus-2
SIRT-1	Sirtuin-1
SMA	smooth muscle actin
SMC	structural maintenance of chromosomes complex
SOD	superoxide dismutase
TBARS	thiobarbituric acid reactive substances
TGF1β	transforming growth factor
TIMP-1	tissue inhibitor of metalloproteinase-1
TLR4	toll-like receptor 4
TNF-α	tumor necrosis factor-α
TUNEL	terminal deoxynucleotidyl transferase dUTP nick end labeling
VCAM-1	vascular cell adhesion molecule-1
VE	minute ventilation
VEGF	vascular endothelial growth factor
VEGFR2	vascular endothelial growth factor receptor 2
VILI	ventilatory-induced lung injury
XEN gas	2.4% H_2_ in air
ZO-1	tight junction protein ZO-1/zonula occludens-1
